# The Role of Genetic Polymorphism and Other Factors on Clopidogrel Resistance (CR) in an Asian Population with Coronary Heart Disease (CHD)

**DOI:** 10.3390/molecules26071987

**Published:** 2021-04-01

**Authors:** Mohammed Ahmed Al-Kaif, Nur Aizati Athirah Daud, Abubakar Sha’aban, Mei Li Ng, Muhamad Ali Sk Abdul Kader, Dzul Azri Mohamed Noor, Baharudin Ibrahim

**Affiliations:** 1 School of Pharmaceutical Sciences, Universiti Sains Malaysia, Penang 11800, Malaysia; mohammed.alkaif@student.usm.my (M.A.A.-K.); aizati@usm.my (N.A.A.D.); abuushaaban@usm.my (A.S.); dzulazri@usm.my (D.A.M.N.); 2 Advanced Medical and Dental Institute, Universiti Sains Malaysia, Penang 13200, Malaysia; nmeili@usm.my; 3 Department of Cardiology, Penang General Hospital, Pulau Pinang 10990, Malaysia; mdali_sheikh@hotmail.com; 4 Faculty of Pharmacy, Universiti Malaya, Kuala Lumpur 50603, Malaysia

**Keywords:** clopidogrel 2, antiplatelet 3, clopidogrel resistance 4, *CYP2C19* polymorphism 5, personalized medicine

## Abstract

Clopidogrel is a widely-used antiplatelet drug. It is important for the treatment and prevention of coronary heart disease. Clopidogrel can effectively reduce platelet activity and therefore reduce stent thrombosis. However, some patients still have ischemic events despite taking the clopidogrel due to the alteration in clopidogrel metabolism attributable to various genetic and non-genetic factors. This review aims to summarise the mechanisms and causes of clopidogrel resistance (CR) and potential strategies to overcome it. This review summarised the possible effects of genetic polymorphism on CR among the Asian population, especially CYP2C19 *2 / *3 / *17, where the prevalence rate among Asians was 23.00%, 4.61%, 15.18%, respectively. The review also studied the effects of other factors and appropriate strategies used to overcome CR. Generally, CR among the Asian population was estimated at 17.2–81.6%. Therefore, our overview provides valuable insight into the causes of RC. In conclusion, understanding the prevalence of drug metabolism-related genetic polymorphism, especially *CYP2C19* alleles, will enhance clinical understanding of racial differences in drug reactions, contributing to the development of personalised medicine in Asia.

## 1. Introduction

Decreased response to clopidogrel among the Asian population is typical due to genetic polymorphism and other factors associated with clopidogrel resistance, estimated to be 70% in some Asian societies. Studying the Asian population is necessary, especially since many Asians have moved to all parts of the world due to increased immigration, making the current guidelines for genetic testing or platelet response testing not generally applicable before prescribing clopidogrel. Therefore, it is essential for clinicians treating Asian patients to consider inter-individual variability in response to clopidogrel when prescribing the drug [1].

Current guidelines suggest the use of dual antiplatelet therapy (DAPT), involving aspirin with a P2Y12 receptor inhibitor to prevent atherothrombotic events in patients with acute coronary syndrome (ACS) and those undergoing percutaneous coronary intervention (PCI) [2,3,4]. Clopidogrel is currently one of the most widely used P2Y12 receptor inhibitors [4,5,6]. Many large clinical trials have confirmed the antiplatelet effect of clopidogrel. One such trial found the use of aspirin in conjunction with clopidogrel antiplatelet therapy in patients with ACS, can reduce adverse coronary events by 20% [7]. This finding agrees with the Clopidogrel and Metoprolol in Myocardial Infarction Trial/Second Chinese Cardiac Study (COMMIT/CCS-2) research that showed that the use of clopidogrel (75 mg) in conjunction with aspirin in a group of myocardial infarction patients was found to reduce major vascular events and mortality [8].

Although clopidogrel has a significant effect on antiplatelet aggregation, studies have shown that platelets’ response to clopidogrel varies significantly between people [9,10]. Some patients can also develop recurrent ischemic events such as stent thrombosis and myocardial infarction during treatment with clopidogrel. This failure of platelet aggregation inhibition in clopidogrel users is referred to as clopidogrel resistance (CR) or platelet hyperresponsiveness [11,12,13]. Studies have shown that 4 to 30% of patients have CR, and 5 to 6% of patients have DAPT resistance after implanting the stent [14,15]. Matetzky et al. (2004) also found that up to 25% of patients with severely raised acute ST-segment elevation myocardial infarction demonstrated CR, which is associated with a higher risk of developing cardiovascular disease [16]. Muller et al. (2003) found that 4.7% of patients who take clopidogrel after PCI have low platelet inhibition rates, which is associated with an increased risk of clinical thrombosis [17]. Based on several studies, the prevalence of CR in the Asian population was reported to be between 17.2 to 81.6%. (Table 1).

Various genetic and non-genetic factors affect clopidogrel resistance. This review aims to summarise the mechanisms and causes of clopidogrel resistance (CR) and the potential strategies to overcome it.

## 2. The Pharmacological Effects of Clopidogrel

**Figure 1:**
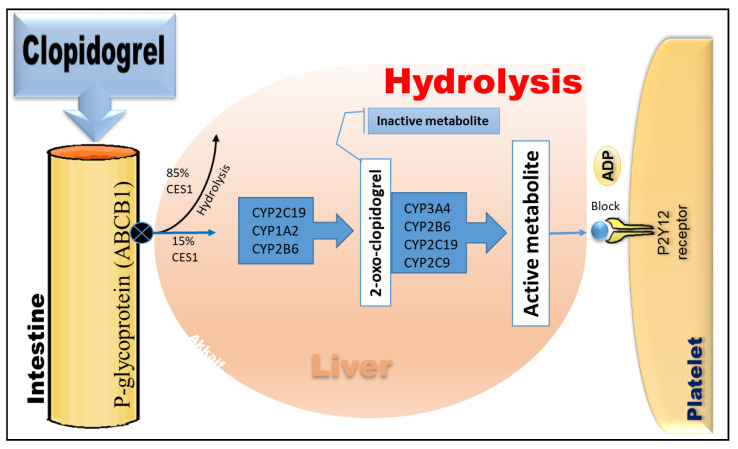
The metabolic pathway of clopidogrel and its target receptors.

Clopidogrel is a thiophene pyridine prodrug that needs to be absorbed by the intestines and transformed into active components by the metabolism of various enzymes in the liver to exert its platelet anti-aggregation effect [26]. The P-glycoprotein (P-gp), encoded by the *ABCB1* gene, regulates clopidogrel absorption in the small intestine [27]. P-gp is a transmembrane protein with the primary function of pumping the drug out of the cell and into the blood circulation. This pumping mechanism may affect the drug’s bioavailability. After clopidogrel absorption in the intestine, 85% is hydrolysed by carboxylesterase 1 (CES1) to inactive metabolites and excreted in urine or faeces [28]. Only 15% is passed through cytochrome P450 in the liver, where clopidogrel is first converted into intermediate metabolite (2-oxo-clopidogrel) by CYP2C19, CYP1A2, and CYP2B6 and then catalysed by CYP2C19, CYP2C9, CYP3A4, and CYP2B6 to produce an active metabolite. It selectively and irreversibly binds to the adenosine diphosphate (ADP) P2Y12 receptor on the platelet membrane to reduce ADP. The binding site blocks the ADP-mediated binding of fibrinogen to its receptor glycoprotein IIb/IIIa, inhibits platelet activation and aggregation, and exerts antiplatelet effects [29,30]. The metabolic pathway of clopidogrel is depicted in Figure 1.

## 3. Definition of Clopidogrel Resistance

There is currently no uniform definition of CR, but the most accepted is that the drug has lost its target of the action. It is generally believed that CR means that a patient still has a thrombotic event after receiving clopidogrel treatment, and laboratory tests show that platelet function is not inhibited [31]. Some researchers refer to it as clinical resistance among patients who have experienced thromboembolism and other adverse events following long-term oral clopidogrel therapy [1]. The incidence of CR varies among different regions and races. According to literature reports, the incidence of CR in Western countries is 5 to 44%, while in Asian populations, it may be as high as 20 to 65% [1,32].

There are several methods commonly used to evaluate platelet function. The oldest and more accurate way is optical turbidimetry, which is often considered the gold standard. This method assesses the responsiveness of platelets to ADP through the function of P2Y1 and P2Y12 receptors. However, because of the repetition rate and the lack of a specific P2Y12 pathway, its use is limited. At present, vasodilator-stimulated phosphoprotein (VASP) phosphorylation assay (VerifyNow) and bedside monitoring are widely used due to the relatively easy operation [11,33]. Tantry and colleagues (2014), in their follow-up studies on CR, confirmed that the available evidence does not support routine screening for hypo/non-responsiveness in patients who started treatment with clopidogrel [34]. So far, there is still a lack of standard experimental methods for diagnosing CR. Clinically, platelet function can be tested to determine the patient’s platelet reaction after medication intake to identify the potential risk of increased cardiovascular or bleeding events. The incidence of CR in elderly patients may be higher than that in younger patients, and the risk of bleeding with clopidogrel is also increased [35,36,37].

The use of platelet function tests (PFTs) to allocate a better selection of antiplatelet drugs to patients with cardiovascular disease has been discussed over the past ten years [38]. These studies mitigated the escalation of antiplatelet therapy according to the results of PFTs for potential clinical benefit. Furthermore, the 2011 American College of Cardiology/American Heart Association guidelines issued a Class IIb recommendation for the use of PFTs among patients taking P2Y12 inhibitors [39]. Still, this classification was downgraded to a Category III recommendation in 2016 [40]. In ACS cases, the latest European guidelines indicate that de-escalation, but not escalation, of P2Y12 inhibitors directed by PFT, with a Class IIb rating, can be considered [41].

## 4. Factors Associated with CR

The mechanism of CR is still unclear. Relevant studies have shown that CR may be influenced by various factors such as race, age, weight, genetic polymorphism, drug interaction, diabetes, inflammation, immature platelets, atherosclerosis, medication compliance and other factors. Despite these various contributing factors of clopidogrel resistance, the exact mechanism is currently unknown [1,37,42,43,44,45,46,47,48,49].

### 4.1. Gene Polymorphism

Many studies have been done to determine the relationship between P2Y12 receptor gene polymorphism and CR (Table 2). Zoheir et al. (2013) found that P2Y12 receptor gene polymorphism is closely related to platelet activity [50]. The P-gp encoded by *ABCB1* regulates the absorption of clopidogrel in the intestines. Earlier studies by Mega et al. (2009) found that *ABCB1* gene polymorphism affects the degree of platelet inhibition, which is closely related to the risk of major adverse cardiac events (MACE) [5]. However, in recent years, studies on the Chinese population have shown no association between *ABCB1* gene polymorphism and CR [51,52,53].

CYP3A4/5 are among the essential enzymes in clopidogrel activation. Previously, Lau et al. (2004) have shown that lower CYP3A4 activity, determined using an erythromycin breath test, is associated with a lower antiplatelet effect of the drug [54].

One study aimed to determine the effect of the *CYP3A* homologs of sub-enzymes (allelic variants of CYP3A4 * 22 and CYP3A5 * 3) on the efficacy of clopidogrel in patients with ACS undergoing percutaneous coronary intervention. The study results found that *CYP3A4 / 5* activity was not associated with platelet aggregation rates, as well as the genotyping and phenotyping of *CYP3A4 / CYP3A5* did not predict the antiplatelet effect of clopidogrel. The researcher recommended more extensive research to prove its clinical relevance [55].

The genetic variations in CYP450 isoenzymes genes (*CYP1A2, CYP2B6, CYP2C9, CYP2C19,* and *CYP3A4*), which are involved in drug metabolism, can influence the variation of pharmacodynamic response to clopidogrel, especially the genetic variation in the CYP2C19 isoenzyme. This enzyme contributes significantly to the two sequential oxidative steps in the biotransformation of clopidogrel into active metabolites [56,57]. Hence, genetic polymorphism of *CYP2C19* could play a crucial role in wide inter-individual and inter-ethnic variabilities in clinical response towards clopidogrel [58,59,60,61].

The choice of antiplatelet therapy (clopidogrel, ticagrelor, or prasugrel) based on individual patient characteristics, such as treatment choice based on genetic data related to clopidogrel metabolism as well as considerations regarding the clinical features of patients may result in a significantly lower rate of ischemic and hemorrhagic events compared to usual practice [70]. The choice of antiplatelet therapy based on both *CYP2C19* gain of function (GOF) and loss of function (LOF) alleles appears to be a preferred approach over universal clopidogrel and universal variant P2Y12 inhibitor therapy for ACS patients with PCI [71,72]. *CYP2C19*-guided escalation and de-escalation are common as clopidogrel persistence in nonfunctional allele carriers is associated with adverse outcomes [73].

Genetic polymorphisms in *CYP2C19* were classified into groups and referred to as alleles. The preliminarily identified alleles include 36 alleles such as CYP2C19 *1,*2, *3, *4, *5, *6, *7 or *8 etc. of which the most significant impact on clopidogrel is *2/*3 mutation sites (weak metabolites) and *17 mutation sites (strong metabolites). The frequency of other variations in most population groups is low [74]. According to clinical guidelines issued by the Clinical Pharmacogenetics Implementation Consortium (CPIC), genotype-related individual variability in metabolic enzyme function is divided into four predicted *CYP2C19* metabolic phenotypes: Poor metabolisers (PMs), intermediate metabolisers (IMs), Extensive metabolisers (EMs), and Ultrarapid metabolisers (UMs) [75] (Table 3).

Many studies have reported wide inter-ethnic variability in *CYP2C19* polymorphism. Asian populations (~ 55.0 to 70.0%) have a higher prevalence rate of *CYP2C19* LOF variant alleles (CYP2C19 *2 and *3) as compared with white populations (~ 25.0 to 35.0%) and black populations (~35.0 to 45.0%) [76,77]. On the other hand, Asian populations (~4.0%) have a low prevalence of the *CYP2C19* GOF variant allele (CYP2C19 *17) as compared to white populations (~18.0%) [78,79].

**Figure 2:**
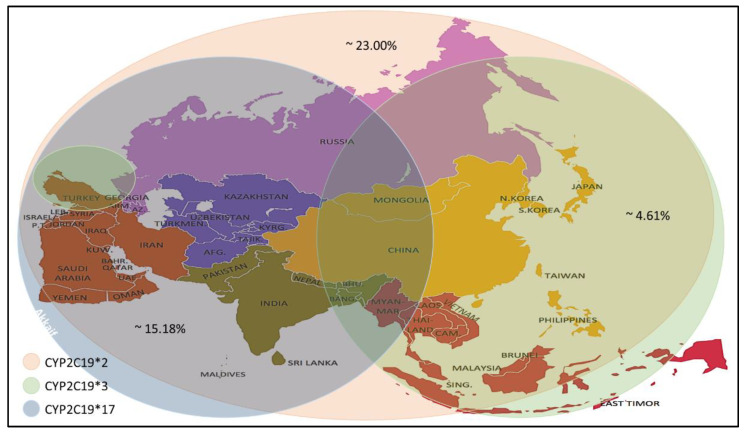
Prevalence of the CYP2C19 * 2/*3/*17 alleles in the Asian population.

Recent studies have reported a variation in the prevalence of individuals carrying *CYP2C19* alleles among the Asian population (Table 4). The CYP2C19 * 2 allele was found in individuals of the selected countries, with prevalence rates ranging between 4.0–59.6%, with an average prevalence rate of 23.00%. The percentage prevalence of CYP2C19 * 2 allele in Saudi Arabia, Qatar and Jordan was less than 10% (residents of the Arabian Peninsula), which is low compared to others. Meanwhile, the CYP2C19 * 3 allele prevalence was found at rates up to 0–13.03% with an average prevalence rate of 4.61%. It is noticed that the spread of this allele is higher in the countries of Southeast and East Asia. Still, its prevalence rates are lower in India, located in the south of Asia, Russia, which is in its north and most countries in West Asia, excluding Turkey. From the CYP2C19 * 17 allele prevalence data, it is noticed that the prevalence rates ranged between (1- 28.72) %, with an average rate of 15.18%, as it is seen here that there are high prevalence rates in the North, South and West Asia. Medium to low rates are observed in some Central and Southeast Asia (Figure 2).

In general, the high allele frequency of CYP2C19 * 2 and * 17 in the Asian population led to the recommendation of a pre-treatment test to monitor for clopidogrel response, dose and to avoid adverse drug reactions after treatment.

### 4.2. Drug Interactions

**Figure 3:**
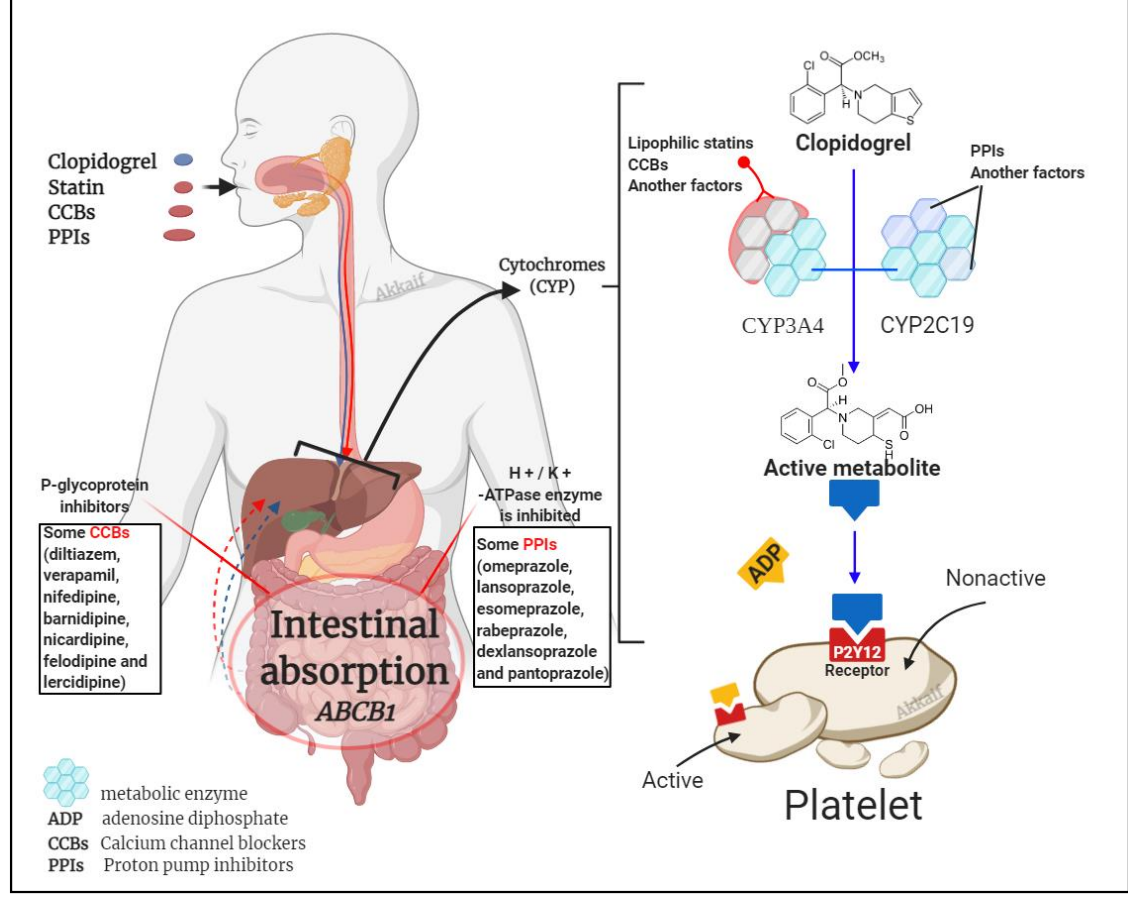
Drug interaction mechanism of clopidogrel with statins, calcium channel blockers (CCBs) and proton pump inhibitors (PPIs).

It is known that clopidogrel is converted into an effective product through the metabolic pathway mediated by CYP enzymes. This process involves a variety of isoenzymes. Such as CYP2C19, CYP3A4, CYP1A2, CYP2C9, etc., but the most important ones are CYP3A4 (~40%) and CYP2C19 (~45%) that contribute to the formation of the active metabolite of clopidogrel; so, the combined use of CYP3A4 and CYP2C19 inhibitors may affect the metabolism of clopidogrel [57,97]. Besides clopidogrel, the CYP3A4 pathway also metabolises statins and calcium channel blockers, and the CYP2C19 pathway metabolises proton pump inhibitors (PPIs) [28,98]. Figure 3 illustrates the mechanism by which these three compounds affect clopidogrel.

#### 4.2.1. Clopidogrel Interaction with Statins

Drug interactions between clopidogrel and statins have been examined and documented over several years. Although most data indicate drug interaction between these drugs, the clinical significance is the determining factor when considering the therapeutic benefit over risk. Statins serve as a lipid-lowering agent, while clopidogrel acts as an inhibitor of platelets. Doctors usually prescribe both drugs to patients for primary prevention of cardiovascular disease and secondary prevention of cardiovascular atherosclerosis (ASCVD) disease among high-risk patients [99,100].

Statins inhibit 3-hydroxy-3-methylglutaryl coenzyme A (HMG-CoA)-reductase, which is responsible for reducing the rate of cholesterol formation. Except for pravastatin, statins undergo extensive hepatic metabolism by multiple cytochrome P450 (CYP) enzymes. The main metabolising enzymes of both clopidogrel and a statin include CYP3A4 and CYP2C9. CYP3A4 mainly metabolises atorvastatin, lovastatin, and simvastatin, whereas CYP2C9 metabolises fluvastatin and rosuvastatin. The level of metabolic activity of CYP3A4 is inversely related to the antiplatelet effects of clopidogrel [28,101].

When statins and clopidogrel are used in combination they may interact via the CYP3A4 metabolic pathway, due to binding site competition. This combination may reduce the antiplatelet activity of clopidogrel, although the effects are still controversial. Lau et al. (2004) had reported that atorvastatin affects clopidogrel level via the CYP3A4 metabolic pathway, while the effect was not present in pravastatin which was not a substrate to CYP3A4 [102]. The report has attracted widespread attention [100,103,104,105]. However, other related studies have not confirmed the effect of CYP3A4 metabolism of statins on the antiplatelet effect of clopidogrel [106,107]. A meta-analysis on the effects of the concomitant administration of clopidogrel and statins reported that statin use decreases patients’ mortality rate with clopidogrel therapy without influencing platelet activation and aggregation [108]. A clinical trial of 190 elective PCI candidates demonstrated that they were already using statins and/or other lipid-lowering agents such as fibrates. The results showed that the administration of a high reload dose of atorvastatin within 24 h prior to the PCI significantly reduced the frequency of myocardial infarction [109]. A study by Karaźniewicz-Łada et al. was the first study identifying the effect of atorvastatin and rosuvastatin on the pharmacokinetics of clopidogrel and its metabolites, and the report had confirmed that systemic exposure to clopidogrel in patients after coronary stent implantation did not depend on statins [110].

#### 4.2.2. Calcium Channel Blockers

Calcium channel blockers (CCBs) are frequently used in patients with high blood pressure, CAD, and arrhythmias. CCBs are metabolized by CYP3A4 to inactive metabolites [111,112]. This may affect the metabolism of clopidogrel via *CYP3A4*, which is a secondary metabolic enzyme for clopidogrel [113]. Recently, researchers have been interested in the interaction of clopidogrel with CCBs and its effect on clopidogrel efficacy, both in vitro and in vivo [114]. Lee et al., 2020 had indicated that CCBs metabolised by *CYP3A4* could reduce the effectiveness of clopidogrel, which is reflected in platelet inhibition. However, the findings on drug interactions between CCBs and clopidogrel are controversial. Amlodipine, which is metabolised by CYP3A4 but not a substrate to P-gp, has been shown to cause alterations in clopidogrel response. Conversely, the co-administration of clopidogrel and verapamil/diltiazem has not been shown to impair the antiplatelet effects induced by clopidogrel. These different results may be explained by the presence or absence of an inhibitory effect of P-gp. P-gp inhibited by CCBs could increase clopidogrel plasma concentration and may attenuate the effect of the interaction between clopidogrel and CCBs through CYP3A4. However, there is no firm evidence that this potential drug interaction between amlodipine and clopidogrel affects clinical outcomes [115].

#### 4.2.3. Proton Pump Inhibitors (PPIs)

PPIs are primary medications that do not require enzyme activity to convert them to their active metabolites. In parietal stomach cells, the H+ / K+ -ATPase enzyme is inhibited, reducing stomach acid production. This drug class includes omeprazole, lansoprazole, esomeprazole, rabeprazole, dexlansoprazole, and pantoprazole. CYP2C19 and CYP3A4 are mainly involved in transforming the PPIs into inactive metabolites [116].

It has been inconclusive for patients who use PPI and clopidogrel simultaneously whether the combination of these two would impact the clopidogrel response. Although several studies have shown no interaction between clopidogrel and PPIs, several questions have been raised about why the antiplatelet inhibition of clopidogrel is reduced with PPIs. Studies published between 2012 to 2016 found that this combined use was associated with significantly higher adverse cardiac events such as major adverse cardiovascular events (MACEs) and ST-Elevation Myocardial Infarction (STEMI) after PCI; however, long-term mortality is not related [117]. It led to a black box warning admonition by the US Food and Drug Administration (FDA) and the European Medicines Agency (EMA) in 2009–2010. Importantly, with each PPI, pharmacokinetics/pharmacodynamics tests have shown that drug-drug interactions are different between clopidogrel and PPIs. Therefore, this is not a class effect but a drug-specific effect involving agents that interfere mainly with the action of *CYP2C19*. The FDA labels were changed according to individual PPIs in 2011–2012, which warn against the concomitant use of omeprazole and esomeprazole with clopidogrel and to highlight the lack of interaction between pantoprazole, lansoprazole and dexlansoprazole with clopidogrel [115].

### 4.3. Dose Factors

The anti-platelet effect of clopidogrel is dose-dependent [17]. The 300 mg loading dose of clopidogrel reaches a steady state after 4 to 24 h. If there is no load, it takes 4 to 7 days to reach a steady-state [118]. Allier et al. found that the antiplatelet effect of clopidogrel 600 mg administered for the first time was equivalent to that of long-term 75 mg patients. Clopidogrel 600 mg administered during long-term treatment can further inhibit platelet aggregation [119]. Due to the increase in thrombus load before treatment, the standard loading dose is not enough to achieve effective platelet inhibition for patients with severe symptoms. Therefore, CR will still occur with conventional-dose treatment [120].

### 4.4. Other Factors

Among other factors, patients’ compliance also directly affects the effectiveness of clopidogrel. Other than that, the antiplatelet effect of clopidogrel is limited in type 2 diabetes patients because this disease is often associated with atherosclerotic disease manifestations; clopidogrel is commonly used in these patients [121]. Diabetes is also a risk factor for reduced antiplatelet effects by clopidogrel [121,122]. There is also a vital relationship found between the level of inflammatory factors and CR caused by abnormal platelet function [123,124,125].

## 5. Strategies to Overcome CR

### 5.1. Increase the Dose of Clopidogrel

Increasing the dose can increase the biological effect of clopidogrel and reduce the incidence of CR. Simultaneously, large doses of clopidogrel can reduce patients’ platelet aggregation rate with CR [126]. For PCI patients, the 600 mg loading dose has a faster response than the 300 mg loading dose and has a more substantial platelet inhibitory effect. In this way, the incidence of CR is significantly reduced [127,128]. At the same time, studies have shown that CR or platelet hyperresponsiveness is still common after the administration of clopidogrel 600 mg load, but increasing the dose can reduce the risk of death from cardiovascular disease, myocardial infarction, and stent thrombosis [5]. In patients with stable coronary heart disease, CYP2C19*2 heterozygous carriers taking 225 mg of clopidogrel per day were shown to achieve the same antiplatelet effect with *CYP2C19* wild-type patients taking 75 mg of clopidogrel per day. In contrast, CYP2C19*2 homozygous patients cannot achieve the desired antiplatelet effect even if they take the 300 mg clopidogrel maintenance dose [129]. CR in patients treated with PCI between high maintenance dose (150 mg · d ^−1^) than conventional maintenance dose (75 mg · d ^−1^) can more effectively prevent major adverse cardiac events (MACE). In the 1-month follow-up after PCI, the incidence of in-stent thrombosis was lower among the group receiving 150 mg · d ^−1^ as compared to the group receiving 75 mg · d ^−1^ (1.1% and 4.9%, *p* = 0.03). Simultaneously, cardiovascular events incidence was also significantly lower in the group with higher doses (2.7% and 7.6%, *p* = 0.03) [130]. However, some studies have shown that high-dose clopidogrel after PCI did not reduce the mortality of cardiovascular events or stent thrombosis incidence than standard doses [131]. Moreover, high-dose clopidogrel may lead to an increased probability of bleeding complications; therefore, the use of high-dose clopidogrel maintenance treatment to avoid treatment resistance requires further research.

### 5.2. Combined Use of Other Antiplatelet Drugs

Ainetdinova et al. [132] found that the probability of resistance to aspirin, clopidogrel, and the combination of these two drugs were 25.7%, 17.1%, and 5.7%, respectively. Therefore, DAPT with aspirin and clopidogrel was shown to reduce the occurrence of drug resistance. Another potential combination therapy uses the GPIIb/IIIa receptor antagonists (such as abciximab, tirofiban and eptifibatide), which can directly block the final pathway of platelet activation, adhesion, and aggregation. Based on clopidogrel therapy, the combined use of GPIIb/IIIa receptor antagonists can further inhibit platelet aggregation [133,134].

### 5.3. Replacement of New P2Y12 Receptor Antagonists

The new P2Y12 inhibitors, ticagrelor and prasugrel, will substantially reduce platelet hyperresponsiveness and improve clinical outcomes relative to the regular clopidogrel dose. Most patients who do not respond to clopidogrel can significantly inhibit the platelet aggregation rate after switching to prasugrel [135] because prasugrel can better inhibit ADP-induced platelet aggregation, which is faster and stronger than clopidogrel. The longer-lasting antiplatelet effect of prasugrel can significantly reduce the occurrence of ischemic events [136]. On the other hand, ticagrelor does not require liver metabolism and not affected by *CYP2C19* gene polymorphism. It was also shown to significantly reduce mortality related to cardiovascular events, myocardial infarction [137]. A study showed that in STEMI patients undergoing PCI for the first time, a loading dose of 180 mg of ticagrelor was more effective than a loading dose of 600 mg of clopidogrel in reducing microvascular damage [138]. There is also literature mentioning that cangrelor has a powerful platelet inhibitory effect. Its effect may be more significant than clopidogrel. Moreover, its half-life is shorter, does not require liver activation, and is a direct antagonist of P2Y12 [26].

### 5.4. Other Management of CR

Active control of blood sugar in patients with coronary heart disease can reduce the incidence of CR. Avoiding the simultaneous application of other drugs that require CYP metabolisms, such as statins, calcium channel blockers, and PPI, would ensure a better response to clopidogrel therapy.

In a randomised trial of TROPICAL-ACS [139,140], a targeted de-escalation regimen with early switching from prasugrel to clopidogrel was established as an effective alternative treatment strategy in ACS patients. However, the study found that patient age was the primary determinant of outcome after PCI, [141,142], especially when using P2Y12 receptor inhibitors during and after PCI [143,144]. Therefore, TROPICAL-ACS performed a randomised assessment of the effect of age on reducing the escalation of antiplatelet therapy. Significant variation was found among the younger patients who showed an increased net clinical benefit resulting from reduced bleeding complications. These results suggest that targeted de-escalation may be a safe and attractive alternative therapy concept for all ACS patients after PCI, while a significant bleeding benefit could be achieved in younger patients [145].

## 6. Conclusions

Clopidogrel plays an essential role in treating coronary heart disease. However, various factors can affect the response to this drug, such as genetic polymorphism, especially CYP2C19 *2 / *3 / *17 in the Asian population. Although there are many methods for detecting platelet resistance, there is a lack of internationally unified standards and laboratory testing systems. There is also a lack of evidence-based medicine for managing CR. We should continue to explore the influencing factors of clopidogrel resistance and the potential strategies to overcome it. Optimising clopidogrel resistance prevention and treatment strategies is vital for identifying and treating high-risk patients as soon as possible.

## Figures and Tables

**Table 1:** Prevalence of clopidogrel resistance (CR) in various studies in the Asian population.

Investigators	Country	Number of Patients	Clopidogrel Loading Dose (mg)	CR
Ma et al. 2019 [18]	China	441	300	17.2%
Pareed et al. 2020 [19]	India	200	300	32%
Namazi et al. 2012 [20]	Iran	112	600	25.90%
Sahib et al. 2016 [21]	Iraq	127	300	24%
Park et al. 2011 [22]	Korea	114	75/150	46%
Amin et al., 2017 [23]	Malaysia	71	600	38%
Sakr et al., 2016 [24]	Saudi Arabia	49 172 83	75 300 600	81.6% 66.3% 55.4%
Tekkeşin et al. 2016 [25]	Turkish	1.238	600	30.2%
Range				17.2–81.6%

**Table 2:** Genetic polymorphism distribution and allele frequencies in clopidogrel-resistant and non-clopidogrel-resistant groups.

Author	Population	Population Sample	Gene	SNP	Genotype	Allele Frequencies		Total (*n*/%)	*p*-Value
						CR Group (*n*/%)	NCR Group (*n*/%)		
Li et al., 2020 [62]	China	126	CYP2C19*2	rs4244285	GG (*1/*1) GT (*1/*2) TT (*2/*2)	9 (23.1%) 21 (53.8%) 9 (23.1%)	48 (55.2%) 30 (34.5%) 9 (10.3%)	57 (45.2%) 51 (40.5%) 18 (14.3%)	0.001 0.041 0.093
			CYP2C19*3	rs4986893	GG (*1/*1) GT (*1/*3) TT (*3/*3)	27 (69.2%) 10 (25.6%) 2 (5.1%)	75 (86.2%) 11 (12.6%) 1 (1.2%)	102 (80.9%) 21 (16.7%) 3 (2.4%)	0.025 0.070 0.176
Al-Azzam et al., 2013 [63]	Jordan	240	CYP2C19*2	rs4244285	GG (*1/*1) GT (*1/*2) TT (*2/*2)	22 (22.9%) 38 (31.7%) 16 (67.7%)	74 (77.1%) 82 (68.3%) 8 (33.3%)	96(40%) 120(50%) 24(10%)	<0.001
Lee et al., 2009 [64]	Korean	387	CYP2C19*2	rs4244285	GG GA AA	55(49.1%) 40(35.7%) 13(11.6%)	155(56.4%) 93(33.8%) 26(9.5%)	210(54.3%) 133(34.4%) 39(10.1%)	0.287
			CYP2C19*3	rs4986893	GG GA AA	80(71.4%) 31(27.7%) 1(0.9%)	236(85.8%) 37(13.5%) 1(0.4%)	316(81.7%) 68(17.6%) 2(0.5%)	0.001
Amin et al., 2017 [23]	Malaysia	71	CYP2C19*2	rs4244285	GG (*1/*1) GT (*1/*2) TT (*2/*2)	11 (40.7%) 8 (29.6%) 8 (29.6%)	19 (43.2%) 22 (50.0%) 3 (6.8%)	30 (42.3%) 30 (42.3%) 11(15.5)	0.026
Alhazzani, et al., 2017 [65]	Saudi Arabia	50	CYP2C19*2	rs4244285	GG GA + AA	21(84%) 4(16%)	10(40%) 15(60%)	31(62%) 19((38%)	0.001
			CYP2C19*3	rs4986893	GG GA + AA	20(80%) 5(20%)	13(52%) 12((48%)	33(66%) 17(34%)	0.036
Shijun et al., 2014 [66]	China	95	CYP3A4*G1	rs2242480	(GG) (GA + AA)	24 (61.50%) 15 (38.50%)	33 (58.90%) 23 (41.10%)	57 (60.00%) 38 (40.00)	0.798
Namazi, et al. 2012 [20]	Iran	112	*CYP3A5*	rs776746	(*1/*1) (*1/*3) (*3/*3)	-	-	9 (8.00%) 42 (37.50%) 61(54.50%)	>0.05
Al-Husein et al., 2018 [67]	Jordan	280	*CYP3A4*	rs2242480	(*1/*1) (*1/*3+ *3/*3)	80(28.6%) 1 (0.4%)	196 (70%) 3 (1.1%)	276 (98.6%) 4 (1.4%)	>0.9999
			*CYP3A5*	rs776746	(*1/*1) (*1/*3) (*3/*3)	57 (20.4%) 23(8.2%) 119(42.5%)	24(8.6%) 10(3.6%) 47(16.8%)	81(28.9%) 33(11.8%) 166(59.3%)	0.961
Lee et al., 2009 [64]	Korean	387	*CYP3A4*	rs2246709	TT TC CC	42(37.5%) 57(50.9%) 12(10.7%)	103(37.5%) 139(50.5%) 28(10.2%)	145(37.5%) 196(50.6%) 40(10.3%)	0.925
				rs2242480	GG GA AA	74(66.1%) 32(28.6%) 6(5.4%)	172(62.5%) 90(32.7%) 13(4.7%)	246(63.6%) 122(31.5%) 19(4.9%)	0.568
			*CYP3A5*	rs776746	GG GA AA	61(54.5%) 41(36.6%) 6(5.4%)	154(56.0%) 102(37.1%) 12(4.4%)	215(55.6%) 143(37.0%) 18(4.7%)	0.808
Shasha et al., 2020 [68]	China	741	*ABCB1*	rs1045642	GG GA + AA	94(38.5%) 222(70.3%)	161(44.4%) 264(62.1%)	255(34.4%) 486(65.6%)	0.021
Chen et al., 2021 [69]	China	204	*MDR1*	rs 1128503	CC CT TT	12 (24%) 17 (34.7%) 20 (40.8%)	40 (25.8%) 65 (41.9%) 50 (32.3%)	52 (25.5%) 82 (40.2%) 70 (34.3%)	0.521
Li et al., 2020 [62]	China	126	*P2Y12*	rs6809699	GG GT TT	15 (38.5%) 21 (53.8%) 3 (7.7%)	67 (79.3%) 18 (20.7%) 2 (2.3%)	82 (66.7%) 39 (30.9%) 5 (2.4%)	0.000 0.000 0.152
Namazi et al., 2012 [20]	Iran	112		rs2046934	CC CT + TT	-	-	104(92.9%) 8 (7.1%)	>0.05
Lee et al., 2009 [64]	Korean	387		rs2046934	TT TC CC	81(72.3%) 26(23.2%) 4(3.6%)	177(64.4%) 89(32.4%) 8(2.9%)	258(66.7%) 115(29.7%) 12(3.1%)	0.139

CR, clopidogrel resistance; NCR, non-clopidogrel resistance; GG, CC, AA, TT, *1/*1, *2/*2, *3/*3, homozygous; GC, GA, GT, CT, *1/*2, *1/*3, heterozygous.

**Table 3:** The categorisation of the predicted CYP2C19 metabolic phenotypes based on the *CYP2C19* genotypes [75].

Likely Phenotype	Genotypes	Examples of Diplotypes
Ultrarapid metaboliser: Normal or increased activity (−5–30% of patients)	An individual carrying two increased activity alleles (*17) or one functional allele (*1) plus one increased-activity allele (*17)	*1/*17, *17/ *17
Extensive metaboliser: Homozygous wild-type or normal activity (~35–50% of patients)	An individual carrying two functional (*1) alleles.	*1/*1
Intermediate metaboliser: Heterozygote or intermediate activity (~18–45% of patients)	An individual carrying one functional allele (*1) plus one loss-of-function allele (*2-*8) or one loss-of-function allele (*2-*8) plus one increased-activity allele (*17)	*1/ *2, *1/*3, *2/*17
Poor metaboliser: Homozygous variant, mutant, low, or deficient activity (~2–15% of patients)	An individual carrying two loss-of-function alleles (*2-*8)	*2/*2, *2/*3, *3/*3

**Table 4:** *CYP2C19* allele frequencies (* 2, * 3 and * 17) % among Asian ethnic groups.

Author	Population	Population Sample	Method	Allele Frequency (%)		
				CYP2C19*2	CYP2C19*3	CYP2C19*17
Zhong et al., (2017) [80]	China	6686	PCR and DNA Sequencing	31.06	4.61	ND
T. Wang et al., (2020) [81]	China	1129	TaqMan-Real-Time PCR	ND	ND	2.5
(Anichavezhi, Chakradhara Rao, Shewade, Krishnamoorthy, & Adithan, (2012) [82]	India	206	PCR-RFLP	40.2	0	19.2
Dehbozorgi et al., (2018) [83]	Iran	1,229	PCR and DNA Sequencing	21.4	1.7	27.1
Sahib, Mohammed, & Abdul-Majid, (2015) [84]	Iraq	221	PCR and DNA Sequencing	15.2	0.2	19.5
Sugimoto, Uno, Yamazaki, & Tateishi, (2008) [79]	Japanese	265	PCR-RFLP	27.9	12.8	1.13
(Sviri, Shpizen, Leitersdorf, Levy, & Caraco, (1999) [85]	Jewish Israeli	136	PCR-RFLP	15	1	ND
Rjoub et al., 2018 [86]	Jordanian	148	PCR-RFLP	9.8	ND	28.72
Kim, Song, Kim, & Park, (2010) [87]	Korean	271	PCR and pyrosequencing	28.4	10.1	1.5
Amin et al., (2017) [88]	Malaysia	89	PCR and DNA Sequencing	59.6	6.74	ND
Riaz et al., (2019) [89]	Pakistan	1028	ASA-PCR	29.0	ND	23.70
(Ayesh, Al-Astal, & Yassin, (2019) [90]	Palestinian	110	PCR-RFLP	15.5	2.3	ND
Elewa, Ali, & Bader, (2018) [91]	Qatar	129	TaqMan-Real-Time PCR	4	0	10
Mirzaev et al., (2017) [92]	Russia	512	TaqMan-Real-Time PCR	11.25	1.2	22
Al-Jenoobi et al., 2013 [93]	Saudi Arabia	192	PCR and DNA Sequencing	8.2	0	26.9
Sukasem et al., (2013) [94]	Thai	1051	AmpliChip CYP450 test	41.95	13.03	4.30
(Arici & Özhan, (2017) [95]	Turkish	160	PCR-RFLP	12	13	25
Vu et al., (2019) [96]	Vietnam	100	PCR-RFLP	20.5	2.5	1
	Total	13662				
	Average			23.00	4.61	15.18

Population sample: The number of screened individuals. ND: No data.

## Data Availability

No new data were created or analyzed in this study. Data sharing is not applicable to this article.
